# Changes of Development from Childhood to Late Adulthood in Rats Tracked by Urinary Proteome

**DOI:** 10.1016/j.mcpro.2023.100539

**Published:** 2023-03-31

**Authors:** Xuanzhen Pan, Yongtao Liu, Yijin Bao, Youhe Gao

**Affiliations:** Beijing Key Laboratory of Gene Engineering Drug and Biotechnology, College of Life Sciences, Beijing Normal University, Beijing, China

**Keywords:** development, lifetime, urinary proteome, mass spectrometry, rat

## Abstract

To date, studies of development have mainly focused on the embryonic stage and a short time thereafter. There has been little research on the whole life of an individual from childhood to aging and death. For the first time, we used noninvasive urinary proteome technology to track changes in several important developmental time points in a group of rats, covering 10 time points from childhood, adolescence, young adulthood, middle adulthood, and near-death in old age. Similar to previous studies on puberty, proteins were detected and they are involved in sexual or reproductive maturation, mature spermatozoa in seminiferous tubules (first seen), gonadal hormones, decline of estradiol, brain growth, and central nervous system myelination, and our differential protein enrichment pathways also included reproductive system development, tube development, response to hormone, response to estradiol, brain development, and neuron development. Similar to previous studies in young adults, proteins were detected and they are involved in musculoskeletal maturity, peak bone mass, development of the immune system, and growth and physical development, and our differential proteins enrichment pathways also included skeletal system development, bone regeneration, system development, immune system processes, myeloid leukocyte differentiation, and developmental growth. Studies on aging-related changes in neurons and neurogenesis have been reported, and we also found relevant pathways in aged rats, such as regulation of neuronal synaptic plasticity and positive regulation of long-term neuronal synaptic plasticity. However, at all time points throughout life, there were many biological pathways revealed by differential urinary protein enrichment involving multiple organs, tissues, systems, etc. that have not been mentioned in existing studies. This study shows comprehensive and detailed changes in rat lifetime development through the urinary proteome, helping to fill the gap in development research. Moreover, it provides a new approach to monitoring changes in human health and diseases of aging using the urinary proteome.

## Highlights

• Exploration of growth and development stages with urinary protein for the first time.
• Growth and development changes confirmed by existing studies can be found in urine.
• Some unreported changes in developmental stages are revealed for the first time.
• Changes are revealed in more processes of organs, tissues, and systems.
• More processes include growth, development, differentiation, remodeling, and so on.

## Graphical Abstract

**Figure:**
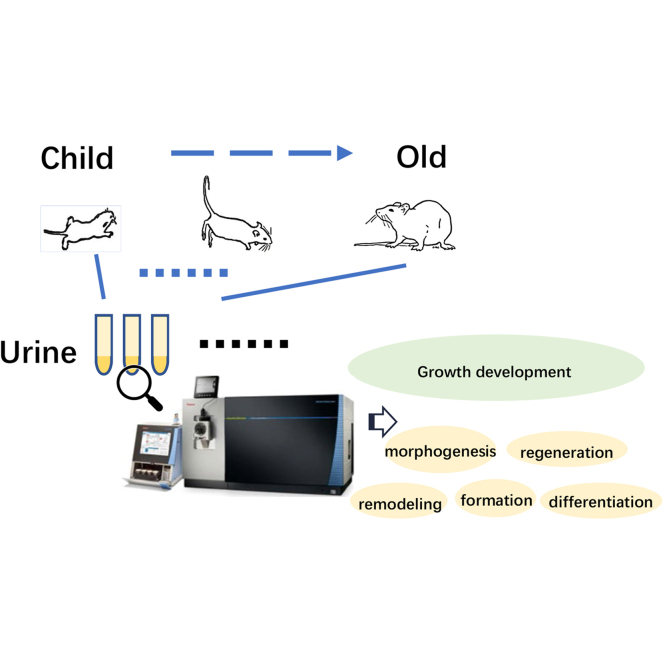

## In Brief

For the first time, we used urinary proteome to track changes in several important developmental time points. Similar to previous studies, differential protein enrichment pathways also included developmental growth. Meanwhile, there were many pathways involving multiple organs, tissues, and systems that have not been mentioned in existing studies. This study shows comprehensive and detailed changes in rat lifetime development, helping to fill the gap in development research. Moreover, it provides a new approach to monitoring changes in human aging.

Urine is considered one of the most valuable biofluids for the discovery of disease biomarkers because urine collection is noninvasive and easy. More importantly, unlike blood, urine is not subject to homeostatic control, and it accumulates small, sensitive, and early changes associated with systemic changes, some of which may be used as biomarkers (1). Urine proteomics has already been applied to various clinical studies (2, 3), including studies on lung cancer (4, 5, 6, 7), breast cancer (8, 9), bladder cancer (10, 11, 12), gastric cancer (13), genitourinary cancer (14), and knee osteoarthritis (15). Moreover, urine-filtered plasma proteins originate from distal organs, including the brain, etc. not only the kidney (16, 17, 18).

Most modern developmental biology research has focused on individuals during pregnancy and a short time before reaching adulthood and has generally focused on a certain organ or system (19, 20). There have also been some studies on elderly individuals (21), but they only involved a certain organ, and individuals of different ages were different, potentially hindering comparability. This study is the first time that the urinary proteome was used to track the whole development of a group of rats from childhood to old age. Urinary proteome can also reflect the overall changes in the body.

## Experimental Procedures

### Experimental Design and Statistical Rationale

The experimental design included rats’ urine samples collected from childhood to near death. Each rat lived in the same environment, which did not change throughout its life. Urine samples were collected at night and stored in a refrigerator at −80 °C. Samples from all time points were processed in the same laboratory environment. We set up seven male rats at each key developmental node as biological duplicates. At the same time, the samples of each rat were repeated twice. All samples were analyzed continuously by the same mass spectrometer over a while. The identified proteins were analyzed to find out differential proteins by comparing adjacent time points or with previous time points based on fold change ≥2 or ≤0.5 criteria, and the statistical significance was defined as a two-sided *p*-value of <0.05(*t* test). These differentials proteins were enriched and analyzed to find the biological pathways’ changes related to growth and development. The differential proteins and enriched pathways identified at each time point also cover at least three rats.

### Acquisition of Experimental Samples and Data

The rat cohort included seven male rats, whose mothers and fathers were born from the same brood of the same parents. We collected samples and data from their major developmental periods including childhood(27 days), adolescence(56 days), youth(170 days, 240 days), adulthood(330 days, 450 days, 600 days), and near death at old age death(800 days, 945 days, 1050 days) (22, 23, 24). All rats were bred from birth to the indicated day, with the same fodders, and they lived in the same environment. The animal experiments were approved by the Ethics Review Committee of the Institute of College of Life Science, Beijing Normal University. Male rats’ parents were purchased from Beijing Charles River Laboratory. The rats were acclimated to the environment for 1 week before the experiment. All experimental animals were utilized following the “Guidelines for the Care and Use of Laboratory Animals” issued by the Beijing Office of Laboratory Animal Management (Animal Welfare Assurance Number: ACUC-A02-2015-004). These urine samples were collected in the same environment, frozen at −80 °C refrigerators, and then processed together.

### Urine Sample Preparation for Label-Free Analysis

After collection, the urine samples were centrifuged at 3000*g* for 30 min at 4 °C and then stored at − 80 °C. For urinary protein extraction, the urine samples were first centrifuged at 12,000*g* for 30 min at 4 °C. Then, 15 ml of urine from each sample was precipitated with three volumes of ethanol at − 20 °C overnight. The pellets were dissolved in lysis buffer (8 mol/L urea, 2 mol/L thiourea, 50 mmol/L Tris, and 25 mmol/L DTT). Finally, the supernatants were quantified using the Bradford assay.

A total of 100 μg of protein was digested with trypsin (Trypsin Gold, Mass Spec Grade, Promega) using filter-aided sample preparation methods (25). Each protein sample was loaded into a 10-kDa filter device (Pall). After washing two times with urea buffer (8 mol/L urea, 0.1 mol/L Tris–HCl, pH 8.5) and 25 mmol/L NH4HCO3 solutions, the protein samples were reduced with 20 mmol/L DTT at 37 °C for 1 h and alkylated with 50 mmol/L iodoacetamide (Sigma) for 45 min in the dark. The samples were then washed with urea buffer and NH4HCO3 and digested with trypsin (enzyme-to-protein ratio of 1:50) at 37 °C for 14 h. The digested peptides were desalted using Oasis HLB cartridges (Waters) and then dried by vacuum evaporation (Thermo Fisher Scientific).

The digested peptides were dissolved in 0.1% formic acid and diluted to a concentration of 0.5 μg/μl. To generate the spectral library for data-independent acquisition (DIA) analysis, a pooled sample (1∼2 μg of each sample) was loaded onto an equilibrated, high-pH, reversed-phase fractionation spin column (84,868, Thermo Fisher Scientific). A step gradient of eight increasing acetonitrile concentrations (5, 7.5, 10, 12.5, 15, 17.5, 20, and 50% acetonitrile) in a volatile high-pH elution solution was then added to the columns to elute the peptides into eight different gradient fractions. The fractionated samples were then evaporated using vacuum evaporation and resuspended in 20 μl of 0.1% formic acid. Two microliters of each fraction were loaded for LC-MS/MS analysis.

### Liquid Chromatography and Mass Spectrometry

The indexed retention time (iRT) reagent (Biognosys) was added at a ratio of 1:10 v/v to all peptide samples to calibrate the retention time of the extracted peptide peaks. For analysis, 1 μg of the peptide from each sample was loaded into a reversed-phase C18 trap column (75 μm x 2 cm, 3 μm, 100 Å) at a flow rate of 0.55 μl/min and then separated with a reversed-phase analytical column (75 μm × 250 mm, 2 μm, C18, 100 Å)with mobile phase A (0.1% formic acid) and mobile phase B (0.1% formic acid in 80% acetonitrile), eluted with a 120-min gradient as follows: 0 min, 3% B; 0 min-3 min, 8% B; 3 min-93 min, 22% B; 93 min-113 min, 35% B; 113 min-120 min, 90% B, and then analyzed with an Orbitrap Fusion Lumos Tribrid Mass Spectrometer (Thermo Fisher Scientific). The LC settings were the same for both the data-dependent acquisition (DDA)-mass spectrometry (MS) and DIA-MS modes to maintain a stable retention time.

For the generation of the spectral library (DIA), the eight fractions obtained from the spin column separation were analyzed with mass spectrometry in DDA mode. The MS data were acquired in high-sensitivity mode. A full MS scan was acquired within a 350 to 1200 m/z range with the resolution set to 120,000. The MS/MS scan was acquired in Orbitrap mode with a resolution of 30,000. The high energy collision dissociation collision energy was set to 30%.

The automatic gain control target was set to 4e5, and the maximum injection time was 50 ms. The individual samples were analyzed in DDA/DIA-MS mode. The variable isolation window of the DIA method with 29 windows was used for DIA acquisition. The full scan was obtained at a resolution of 120,000 with an m/z range from 400 to 1200, and the DIA scan was obtained at a resolution of 30,000. The automatic gain control target was 1e5, and the maximum injection time was 50 ms. The high energy collision dissociation collision energy was set to 35%.

### Mass Spectrometry Data Processing

The MS data of the rat cohort were used to performed label-free quantitative comparisons. Base peak chromatograms were inspected visually in Xcalibur Qual Brower version 4.0.27.19(Thermo Fisher Scientific). To generate a spectral library, 10 DDA raw files were first searched using Proteome Discoverer (version 2.1; Thermo Fisher Scientific) with SEQUEST HT against the UniProt rat sequence database (April 17, 2021; 36,181 sequences). The iRT sequence was also added to the human database. The search allowed two missed cleavage sites in trypsin digestion. Carbamidomethyl (C) was specified as a fixed modification. Oxidation (M) was specified as the variable modification. The parent ion mass tolerances were set to 10 ppm, and the fragment ion mass tolerance was set to 0.02 Da. The Q value (FDR) cut-off at the precursor and protein levels was 1%. Then, the search results were imported into Spectronaut Pulsar 15.7.220308.50606 (Biognosys AG; https://biognosys.com/software/spectronaut/) software to generate the spectral library (26).

The individual acquisition DIA files were imported into Spectronaut Pulsar 15.7.220308.50606 with default settings. And 10 ppm for precursor mass tolerance, 0.6 Da for fragment mass tolerance. The peptide retention time was calibrated according to the iRT data. Cross-run normalization was performed to calibrate the systematic variance of the LC-MS performance, and local normalization based on local regression was used (27). Protein inference was performed using the implemented IDPicker algorithm to generate the protein groups (28). All results were then filtered according to a Q value less than 0.01 (corresponding to an FDR of 1%). The peptide intensity was calculated by summing the peak areas of the respective fragment ions for MS2. The protein intensity was calculated by summing the respective peptide intensities.

**Fig. 1:**
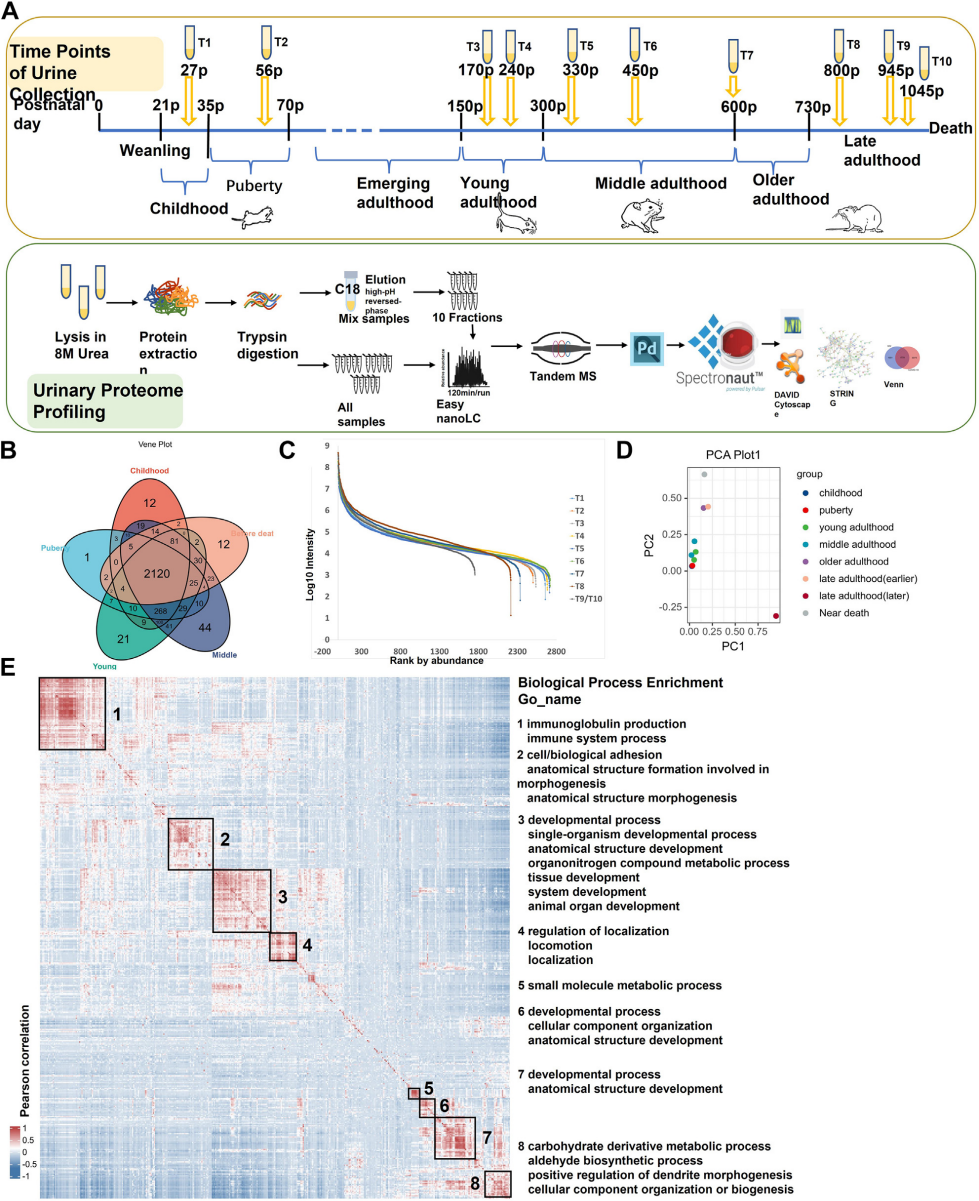
**Urinary proteome profiling of the developing rats.***A*, illustration of rat developmental stages and 15 sampling time points. The experimental procedure and data analysis workflow were below. *B*, the overlap of proteins that were identified in five stages, including childhood, puberty, young adulthood, middle adulthood, and near death. Common proteins were identified among all stages and stages exclusive proteins were also present. *C*, dynamic ranges of the rat urinary proteome were measured at 10 time points. Proteins identified in all stage cohorts were ranked according to their MS signals, which covered more than five orders of magnitude. *D*, principal component analysis of the temporal proteome data, including 10 time points. Time points before middle age were close together. *E*, global correlation map of proteins generated by clustering the Pearson correlation coefficients of all pairwise protein comparisons.

The permutation combination comparison method was used to analyze every rat’s data individually at all time points. The methods included two means: comparing two adjacent time points and comparing one phase time point with the previous phase time point (as shown in Fig. 1). The differential proteins were screened with the following criteria: proteins with at least two unique peptides were allowed; fold change ≥2 or ≤0.5; and *p* < 0.05 by Student’s *t* test. Group differences resulting in *p* < 0.05 were identified as statistically significant. The *p*-values of group differences were also adjusted by the Benjamini and Hochberg method (29). The differential proteins were analyzed by Gene Ontology (GO) based on biological processes (BPs), cellular components, and molecular functions using DAVID (30) and BPs from WebGestalt (http://www.webgestalt.org). Protein interaction network analysis was performed using the STRING database (https://string-db.org/cgi/input.pl) and visualized by Cytoscape (V.3.7.1) (31) and OmicsBean workbench (http://www.omicsbean.cn).

## Results

### Overview of All Rat Cohorts for Urinary Proteome Analysis

All rat cohorts were assessed using label-free DIA LC-MS/MS quantification to characterize the urinary protein profile (32, 33) (Fig. 1*A*). To map proteome changes between individuals at different time points, we analyzed 144 urine samples (including 126 individual samples and 18 mix samples) from rat cohorts. To maximize proteome depth, a spectral library was generated by merging three sublibraries: (i) a library constructed by DDA consisting of eight fractions of pooled urine samples; (ii) a DDA library converted from consisting of 10 fractions of urine samples by proteome discoverer; and (iii) a library generated from the DIA analysis of all analyzed samples. A total of 2960 protein groups were identified in all samples of the rat cohort (supplemental Table S1).

All rat samples were collected from 10 time points. Among them, the first time point(T1) was in childhood, the second time point(T2) was in adolescence, the third and fourth time points(T3, T4) were in young adulthood, the fifth and sixth time points(T5, T6) were in middle adulthood, the seventh time point(T7) was at the beginning of reproductive decline, the eighth time point(T8) was in later life, only three rats were left by the ninth time point(T9), and the 10th time point(T10) was taken 2 days before the death of the last rat(detailed information in Fig. 1*A*). While some proteins were unique in each period, but 2120 proteins were identified in all periods (Fig. 1*B*). The quantified protein intensities spanned five orders of magnitude in all time point cohorts and the top 10 most abundant proteins contributed approximately half of the total urinary proteome signal (Fig. 1*C*). To examine the data quality of all time point samples, principal component analysis was conducted (Fig. 1*D*), in which before middle adulthood, the time points gathered together, while the time points from late adulthood and before death were far apart. The global correlation map contained pairwise relations of all urinary proteins across 126 samples from all time point cohorts. Unsupervised hierarchical clustering of the pairwise Pearson correlation coefficients revealed some small clusters of coregulated proteins (Fig. 1*E*). These clusters were chiefly enriched for proteins with GO)-terms as well as other significant terms. The BPs included immune system processes, development, morphogenesis, localization, and metabolic process.

### The Same Features Between Existing Researches and Urinary Development Research

**Fig. 2:**
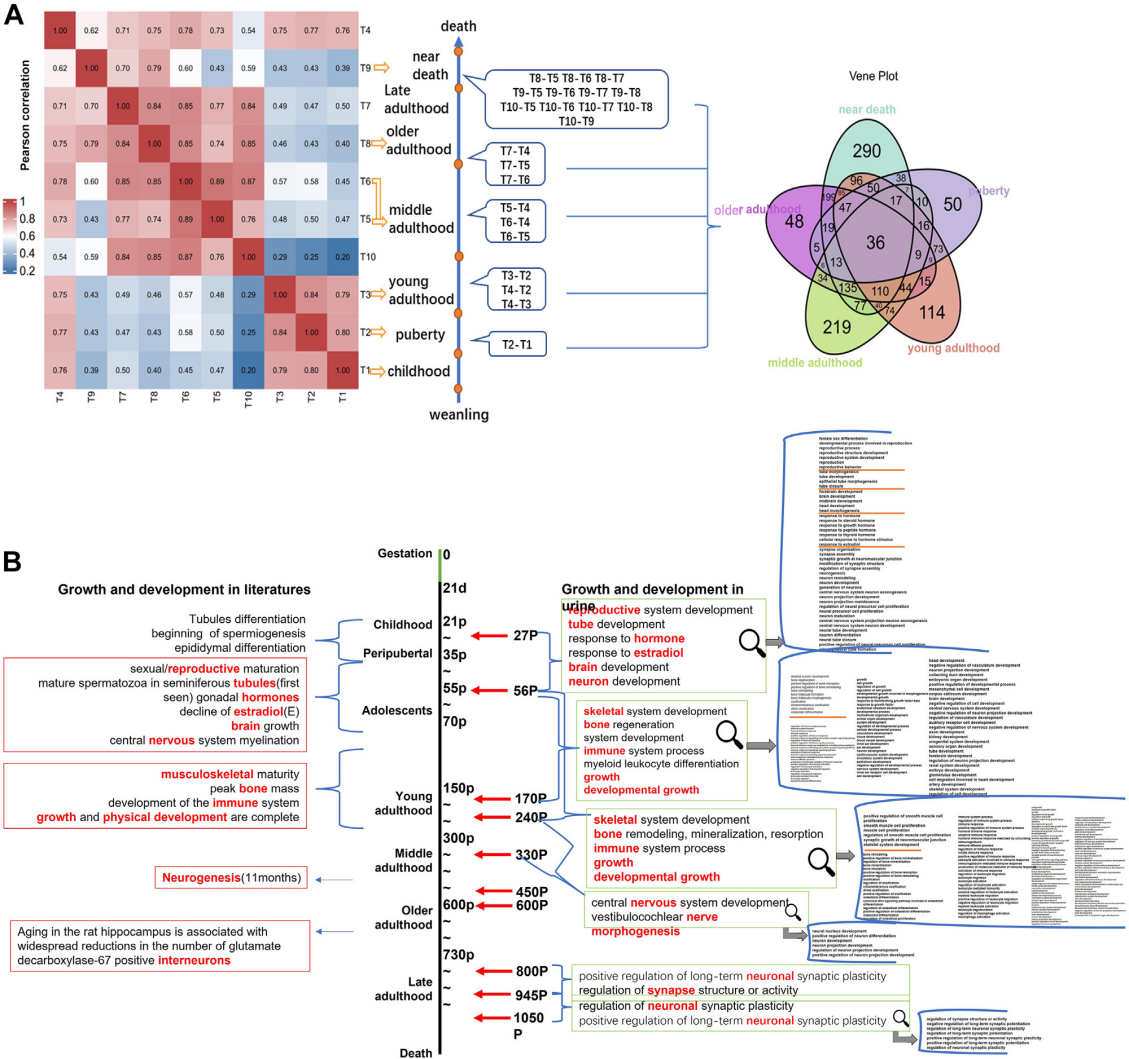
**Comparison between previous researches and urinary development research.***A*, hierarchical clustering analyses of temporal proteome data separated the rat development into six phases. The process of obtaining and analyzing differential proteins was shown. The middle shows the comparison of all time points. The overlap of differential proteins that were identified in five stages was also shown (fold change >2 or <0.5, *p*-value <0.05). *B*, the Panel showed comparisons of previous studies involving various stages of development and urinary development research for mutual validation. The biological pathways displayed met the requirement of *p*-value adjusted <0.01.

Unsupervised hierarchical clustering of Pearson correlation coefficients revealed that most time points were arranged in sequence, which is consistent with development or aging over time (Fig. 2*A*). To achieve higher confidence, differential proteins identified in at least two of the seven biological replicates in at least one time point were used for further statistical analyses. Differential proteins also met the conditions of having *p*-value <0.05 and fold change >2 or <0.5. The differential proteins were compared between the two time points, and all the compared time points were shown in Figure 2*A*. The crossover relationship of differential proteins in the five stages was shown in Figure 2*A*. There were 36 differential proteins in common in the five stages, and many proteins were only produced in certain stages. Four rats died shortly after 800 days, two died shortly after 945 days, and the remaining rat lived for 147 days. Each rat's late development and aging process were different, which reflected certain individual differences.

The differential proteins from samples comparisons between the two time points were analyzed separately for enrichment GO analysis processed by OmicsBean (34). We found that all the biological pathways identified based on the enrichment of urine differential proteins corresponded to pathways identified in previous researches, these pathways could be mutually verified, as shown in Figure 2*B*. As mentioned in previous studies, the peripubertal period occurs from the onset of puberty, when circulating gonadal hormones start to rise, leading to sexual/reproductive maturation (35, 36); while puberty is the developmental stage in which sexual development is completed, and reproductive capacity or fertility is achieved (37, 38); the onset of puberty in male rats is when mature spermatozoa are first seen in seminiferous tubules (35); estradiol increased in peripubertal period in rats, maintaining expression in adolescence (36); significant brain growth is ongoing in the rat until 9 weeks of age and central nervous system myelination in limbic structures is not complete until 6 weeks of age (39, 40, 41). Accordingly, the differential proteins obtained on days 56 and 27 were compared and found to be enriched in similar biological pathways: reproductive system development, tube development, response to hormone, response to estradiol, brain development, neuron development (*p*-value adjusted <0.01, only a few major pathways were listed here, and more detailed information was presented in supplemental Table S2).

Researches on rats that enter adulthood after puberty had found that adulthood in rats is determined according to musculoskeletal maturity (42), and adult life is after growth and physical development are complete (43); unlike humans, bone growth never completely stops in rats (44); peak bone mass is not reached until around 26 weeks of age in rodents (45, 46, 47); the development of the immune system is defined by changes in thymus size and cellular content over early development as well as key immunological markers and T and B lymphocyte production increases over the first 26 weeks of life (48, 49, 50, 51). Similarly, the biological pathways enriched by the differential proteins obtained by comparing young adulthood with adolescence also contained these processes: skeletal system development, bone regeneration, system development, immune system process, myeloid leukocyte differentiation, growth, developmental growth, bone remodeling, mineralization, and resorption.

Some studies had also focused on neurogenesis or interneurons in middle-aged and old-aged rats (21, 52). We also found similar biological pathways, such as central nervous system development, vestibulocochlear nerve morphogenesis, regulation of neuronal synaptic plasticity, and positive regulation of long-term neuronal synaptic plasticity in the comparison between middle adulthood and young adulthood and between late adulthood and older adulthood.

**Fig. 3:**
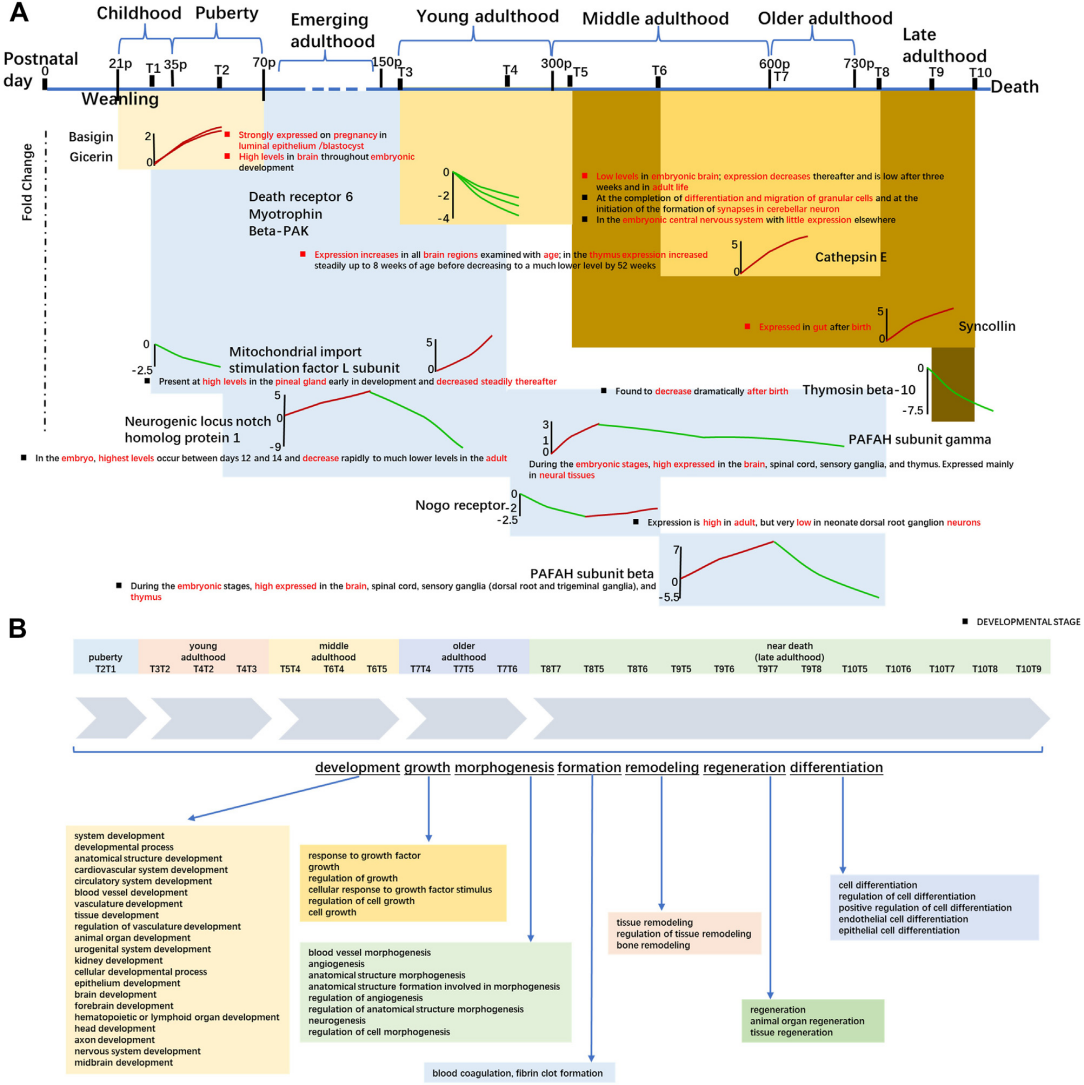
**Overview of the biological pathway analysis of differential protein enrichment obtained by comparison at all time points.***A*, differential proteins involved in one or two developmental stages and their expression changes, as well as their related developmental stages reported in the literature (fold change >2 or <0.5, *p*-value <0.05). *B*, biological pathways involved many organs, tissues, or systems and spanned many biological processes such as development, growth, differentiation, remodeling, etc. Only some major biological processes covering five developmental stages were shown in this figure (*p*-value adjusted <0.01), and the comprehensive biological processes were shown in supplemental Table S3.

We also conducted a literature search for significantly changed differential proteins and found proteins at various time points in our study that were related to the developmental stages mentioned in previous studies. A total of 40 kinds of differential proteins (*p*-value <0.05 and fold change >2 or <0.5) are involved. As shown in Figure 3*A*, we list some proteins involved in one or two developmental stages and their expression changes, as well as their related developmental stages reported in the literature. The specific proteins and descriptions of developmental stages related to the literature were shown in supplemental Table S3.

### Presentation of Developmental Stages in the Urinary Proteome

Enrichment analysis was performed on the differential proteins (fold change >2 or <0.5, *p*-value <0.05) obtained by pairwise comparison at adjacent time points in each stage. As shown in Table 1, biological pathways are common to at least three stages (*p*-value adjusted <0.01, PAS Z-score means the pathway activation strength Z-score, which served as the activation profiles of the Signaling pathways based on the expression of individual genes). Among them, all time points had in common tissue development and system development. Anatomical structure development/morphogenesis, blood vessel development/morphogenesis, cardiovascular system development, circulatory system development, response to hormone, epithelium development, animal organ development, angiogenesis, regulation of growth, urogenital system development, immunoglobulin production, response to steroid hormone, response to peptide hormone, cellular response to growth factor stimulus, epithelial cell differentiation, positive regulation of cell differentiation, positive regulation of cell proliferation, regulation of vasculature development also involved five stages (More detailed information for all time points was presented in supplemental Table S2). And most of these pathways were not mentioned in previous researches.

**Table 1:** Enrichment analysis of the differential proteins in five stages

Go_name(BP) PAS_Zscore	Puberty	Young Adulthood		Middle Adulthood		Older Adulthood	Near death (late adulthood)			no.Zscore >0	no.Zscore <0
Time point	T2T1	T3T2	T4T3	T5T4	T6T5	T7T6	T8T7	T9T8	T10T9	**↑**	↓
tissue development	3.21	2.85	−2.56	−1.98	1.97	2.95	2.21	1.89	−1.83	6	3
system development	6.01	−6.47	−5.21	−4.93	4.8	6.32	5.49	4.71	−4.64	5	4
single-organism developmental process	7.74	3.46	6.12	−6.46	5.8	7.53	6.58	/	−6.3	6	2
developmental process	7.92	3.46	2.66	−6.49	5.8	7.58	6.58	/	−6.3	6	2
anatomical structure development	7.31	3.37	5.92	−6.23	5.57	7.01	6.22	/	−5.73	6	2
vasculature development	1.12	−1.75	/	−0.79	0.98	1.4	0.75	1.48	−1.07	5	3
blood vessel morphogenesis	0.96	−1.3	/	−0.72	0.92	1.26	0.7	1.2	−0.93	5	3
blood vessel development	1.12	−1.7	/	−0.79	0.92	1.4	0.75	1.48	−1.07	5	3
cardiovascular system development	1.55	−1.87	/	−0.83	1.12	1.78	1.05	1.55	−1.15	5	3
circulatory system development	1.61	−1.93	/	−0.91	1.16	1.78	1.1	1.6	−1.2	5	3
anatomical structure morphogenesis	3.82	−3.99	2.7	−3.27	/	3.79	3.14	2.74	−3.05	5	3
regulation of multicellular organismal development	2.64	−3.06	−2.67	−1.92	/	2.77	2.1	2.27	−2.25	4	4
regulation of developmental process	3.45	−0.24	−3.36	−2.79	/	3.19	2.8	2.77	−2.89	4	4
response to hormone	1.72	/	1.97	−2.04	1.56	1.79	1.62	1.13	/	6	1
multicellular organism development	6.45	−6.9	−5.33	/	5.05	6.51	6.03	4.81	/	5	2
epithelium development	1.72	1.72	−1.91	−1.43	1.17	1.44	1.43	/	/	5	2
epithelial cell proliferation	0.49	−1	/	−0.44	/	0.83	0.54	0.66	−0.6	4	3
animal organ development	4.65	−5.29	−3.71	−3.84	3.68	4.82	4.28	/	/	4	3
positive regulation of developmental process	1.98	−0.22	−1.85	−1.82	/	1.67	1.5	1.73	/	4	3
anatomical structure formation involved in morphogenesis	1.81	−1.78	−1.7	−1.44	/	2.16	/	1.42	−1.49	3	4
cell proliferation	2.82	−2.36	−2.89	−2.5	/	2.95	−2.48	2.3	/	3	4
angiogenesis	0.77	−1.18	/	/	0.87	1.13	0.63	/	−0.81	4	2
tissue remodeling	0.56	−0.77	/	/	/	0.44	0.2	0.14	−0.27	4	2
regulation of growth	1.03	1.23	−0.79	−0.66	/	0.91	/	0.69	/	4	2
regulation of anatomical structure morphogenesis	1.81	1.79	−0.32	−1.52	/	1.55	/	1.22	/	4	2
regulation of epithelial cell proliferation	0.29	−0.47	/	−0.21	/	0.68	/	0.46	−0.42	3	3
response to growth factor	1.62	−1.46	/	−0.81	/	1.04	/	0.88	−0.95	3	3
growth	1.55	−0.98	−0.32	/	/	1.48	/	1.01	−1.22	3	3
regulation of cell proliferation	2.33	−1.96	−2.41	−2.13	/	2.5	/	2.01	/	3	3
cell differentiation	4.89	−5.32	−4.46	−3.74	/	4.53	4.74	/	/	3	3
cellular developmental process	5.04	−5.53	−4.82	−4.35	/	4.76	4.74	/	/	3	3
urogenital system development	0.27	−0.24	/	−0.5	0.55	−1.27	0.01	/	/	3	3
immunoglobulin production	/	0.61	−0.86	−0.21	/	−0.05	/	−1.39	−0.69	1	5
response to steroid hormone	0.97	/	/	−0.83	/	0.99	0.92	0.49	/	4	1
response to peptide hormone	0.74	/	1.06	−0.74	0.68	/	0.66	/	/	4	1
positive regulation of tissue remodeling	0.06	−0.42	/	/	/	0.12	0.02	/	0.4	4	1
cytokine production	0.77	0.9	−1.32	/	/	0.9	/	0.66	/	4	1
negative regulation of cytokine production	0.38	/	−0.76	/	/	0.41	/	0.38	−0.48	3	2
regulation of tissue remodeling	0.06	−0.48	/	/	/	0.19	−0.18	/	0.42	3	2
cellular response to growth factor stimulus	1.47	−1.29	/	/	/	0.85	/	0.76	−0.89	3	2
epithelial cell differentiation	0.96	1.27	−0.69	−0.59	0.78	/	/	/	/	3	2
positive regulation of cell differentiation	1.45	1.51	−1.76	−1.19	/	/	/	1.03	/	3	2
positive regulation of cell proliferation	1.07	/	−1.61	−1.41	/	1.25	/	1.14	/	3	2
regulation of vasculature development	0.35	−0.22	/	−0.3	/	0.69	/	0.21	/	3	2
kidney development	0.21	0.15	/	−0.29	0.49	−1.1	/	/	/	3	2
aging	0.58	/	/	−0.35	/	−1.17	/	0.45	−1.01	2	3
regulation of cell differentiation	2.33	−2.55	−2.67	−1.83	/	/	2.15	/	/	2	3
regulation of cell growth	0.7	−0.55	−0.31	−0.47	/	/	/	0.31	/	2	3
skin development	0.31	/	−0.37	−0.2	−0.56	/	0.22	/	/	2	3
production of molecular mediator of immune response	/	0.71	−0.89	−0.26	/	/	/	−1.59	−0.73	1	4
endothelial cell proliferation	−0.01	−0.52	/	/	/	−0.18	/	0.17	−0.01	1	4
tube development	0.66	−0.25	−1.19	−0.75	/	−0.85	/	/	/	1	4

Legends:The two decimal digits represent the zscore for each biological pathway.

Legends:The last two column of numbers represent that number of zscore greater or less than 0 for each pathway at each time point.

We compared the obtained differential proteins at the 10 time points for enrichment analysis, respectively. The enriched biological pathways are mostly involved in development, growth, morphogenesis, formation, remodeling, regeneration, differentiation, and others (*p*-value adjusted <0.01). Figure 3*B* shows the biological pathways involved in most stages, and there were many pathways involving fewer stages, as shown in supplemental Table S4. It was found that the enriched biological pathways involved aging and modulation of age-related behavioral decline. We also found pathways that were involved in the growth and development of many organs, tissues, or systems, such as the brain, neurons, bone, kidney, liver, gland, cardiovascular system, hormone, etc. All specific biological pathway information and Z-score of pathways were shown in supplemental Table S4. Among many developmental pathways, we found pathways involved in circulatory system development, including cardiovascular system development, blood vessel development, vasculature development, hematopoietic or lymphoid organ development, regulation of vasculature development, and positive regulation of vasculature development. We also found pathways related to urogenital system development, including renal system development, metanephric loop of Henle development, metanephric proximal tubule development, kidney development, and collecting duct development. Hepatobiliary system development also appeared, including liver development. In addition, the pathways were found to be involved in the gland development, nervous system development, including neuron projection development, neuron development, central nervous system development, axon development, regulation of neuron projection development, positive regulation of neuron projection development, regulation of nervous system development, positive regulation of nervous system development, and neural nucleus development; skin development, including epithelium development, endothelial cell development, epithelial cell development, endothelium development, epidermis development, and skin epidermis development; skeletal system development, including cartilage development, mesenchymal cell development, regulation of osteoclast development, cartilage development, involved in endochondral bone morphogenesis, bone development, osteoclast development, and growth plate cartilage development; animal organ development, including sensory organ development, ear development, inner ear development, inner ear receptor cell development, head development, brain development, glial cell development, substantia nigra development, embryonic organ development, embryo development, reproductive structure development, and reproductive system development, developmental process involved in reproduction, tissue development, connective tissue development. And there were also immune system development, tube development, developmental growth involved in morphogenesis, hair follicle development, biomineral tissue development, stem cell development, camera-type eye development.

## Discussion

In this study, we used the urinary proteome to monitor the growth and development of a batch of rats from childhood to near death for the first time. The urinary proteome comprehensively reflected the growth and development of each stage and covered almost all aspects of the body from organs to tissues to the systems.

In comparison with previous studies, we found many body changes that were not mentioned in previous studies in many stages. We also found changes in some organs with age and aging-related pathways covering almost all time points. Urine collection is noninvasive and convenient. It is believed that this experiment can become a highlight in the field of growth and development as a milestone, demonstrating for the first time that urine can reveal changes in all aspects of body growth and development, providing ideas for monitoring the body conditions of patients, including for clinical prognosis, and for aging research in the future.

## Data Availability

The mass spectrometry proteomics data have been deposited to the ProteomeXchange Consortium (http://proteomecentral.proteomexchange.org) *via* the iProX partner repository (53) with the dataset identifier PXD037207: (http://proteomecentral.proteomexchange.org/cgi/GetDataset?ID=PXD037207

https://www.iprox.cn/page/project.html?id=IPX0005129000).

## Supplemental data

This article contains supplemental data.

## Conflict of interests

The authors declare no competing interests.
